# Oral Deucravacitinib for Treatment-Refractory Cutaneous Lupus Erythematosus

**DOI:** 10.7759/cureus.81539

**Published:** 2025-03-31

**Authors:** Ethan J Matthew, Kritin K Verma, Brent Paulger

**Affiliations:** 1 Dermatology, Texas Tech University Health Sciences Center, Lubbock, USA; 2 Medicine, Texas Tech University Health Sciences Center, Lubbock, USA; 3 Dermatology, Dermatology Associates of West Texas, Lubbock, USA

**Keywords:** cutaneous lupus, deucravacitinib, discoid lupus, subacute cutaneous lupus, tyk2

## Abstract

This case report describes a 66-year-old female patient with a history of treatment-resistant chronic cutaneous lupus and discoid lupus (DL) for 25 years. The patient failed traditional antimalarial therapies for cutaneous lupus and other systemic and topical treatments, including azathioprine, mycophenolate mofetil, methotrexate, and dapsone. The patient was started on deucravacitinib at a daily dose of 6 mg and showed resolution of active lesions at five months, with post-inflammatory hypopigmentation at sites of previous lesions. The patient showed clinical resolution at eight months after starting deucravacitinib. Deucravacitinib is an allosteric inhibitor of TYK2, an enzyme within the JAK/STAT pathway, and may represent a novel therapy in the treatment of cutaneous lupus through the inhibition of type I interferon signal propagation. Further studies of deucravacitinib, including longitudinal evaluation, should be conducted to determine effectiveness within this patient population.

## Introduction

Subacute cutaneous lupus (SCLE) and discoid lupus (DL) are common forms of cutaneous lupus characterized by chronic and relapsing erythematous plaques. Lesions of SCLE often manifest on the sun-exposed areas of the face, scalp, neck, and upper trunk, with DL lesions manifesting on both sun-exposed and sun-sheltered areas [1]. Lesions of both SCLE and DL may resolve with hypopigmentation or hyperpigmentation. Discoid lupus lesions are problematic on cosmetically sensitive areas such as the face and scalp. In these areas, resolution of lupus lesions may be accompanied by atrophy, potentially disfiguring scarring, and alopecia. The foundation of treatment of cutaneous lupus remains hydroxychloroquine sulfate in doses of 200 mg once or twice daily.

The biochemical pathogenesis of cutaneous lupus is complex, with type I interferons playing a critical role in lesional skin. Inhibition of this pathway with deucravacitinib represents a novel approach to the treatment of cutaneous lupus [1,2]. Deucravacitinib, an inhibitor of TYK2, a molecular propagator of type I interferons within the JAK/STAT pathway, represents a new therapeutic approach for controlling cutaneous lupus. Deucravacitinib at this time is FDA approved to treat moderate to severe plaque psoriasis. With limited reports of cutaneous lupus treated with deucravacitinib in the current literature, we present a case of treatment-refractory chronic cutaneous lupus with clinical resolution with deucravacitinib [3-5].

The patient provided informed consent, and a written, signed consent statement was submitted to the journal.

## Case presentation

This is a case of a 66-year-old female patient with a history of treatment-resistant chronic discoid and SCLE for 25 years. The patient had initially presented to the clinic for a rash on the face, scalp, ears, upper trunk, and arms that had been present for six months. On initial presentation, biopsy results were consistent with SCLE. Laboratory values on initial presentation showed no abnormalities of cell counts, urinalysis was without evidence of hematuria, and metabolic panel and sedimentation rates were within normal limits. The antinuclear antibody (ANA) was positive at a titer of 1:320, the anti-SSA/RO antibody was positive, and the rheumatoid factor was negative. The patient denied symptoms of arthritis, pleurisy, mucositis, and neurological disturbance. There were no concerns for systemic lupus erythematosus (SLE) from the managing dermatologist and independent rheumatologist.

Throughout the course of her illness, the patient trialed and failed multiple medications due to side effects, including an allergic reaction to hydroxychloroquine sulfate. The list of trialed systemic medications includes hydroxychloroquine sulfate, azathioprine, mycophenolate mofetil, methotrexate, and dapsone. Topical medications trialed included clobetasol ointment 0.05%, crisaborole ointment 2%, tapinarof 1% cream, ruxolitinib cream 1.5%, and tacrolimus ointment 0.1%. The patient was managed with intralesional triamcinolone injections and multiple courses of oral prednisone for flares throughout the disease course and seen in clinic every four to eight weeks. In the summer months, with increased sun exposure, the patient often required additional courses of oral prednisone. For this duration, no evidence of progression to SLE was noted, and routine laboratory monitoring remained unchanged. Most treatment modalities provided minimal clearance of active lesions. The patient noted moderate improvement with methotrexate 20 mg weekly but stopped due to an urticaria-like rash with headache and bruising.

**Figure 1:**
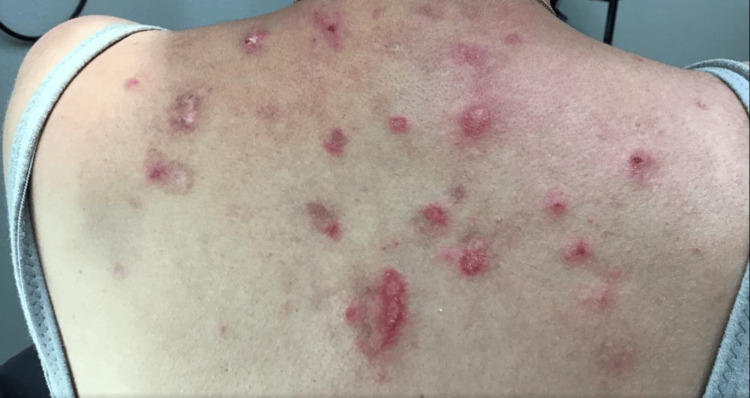
Erythematous plaques on the upper back

**Figure 2:**
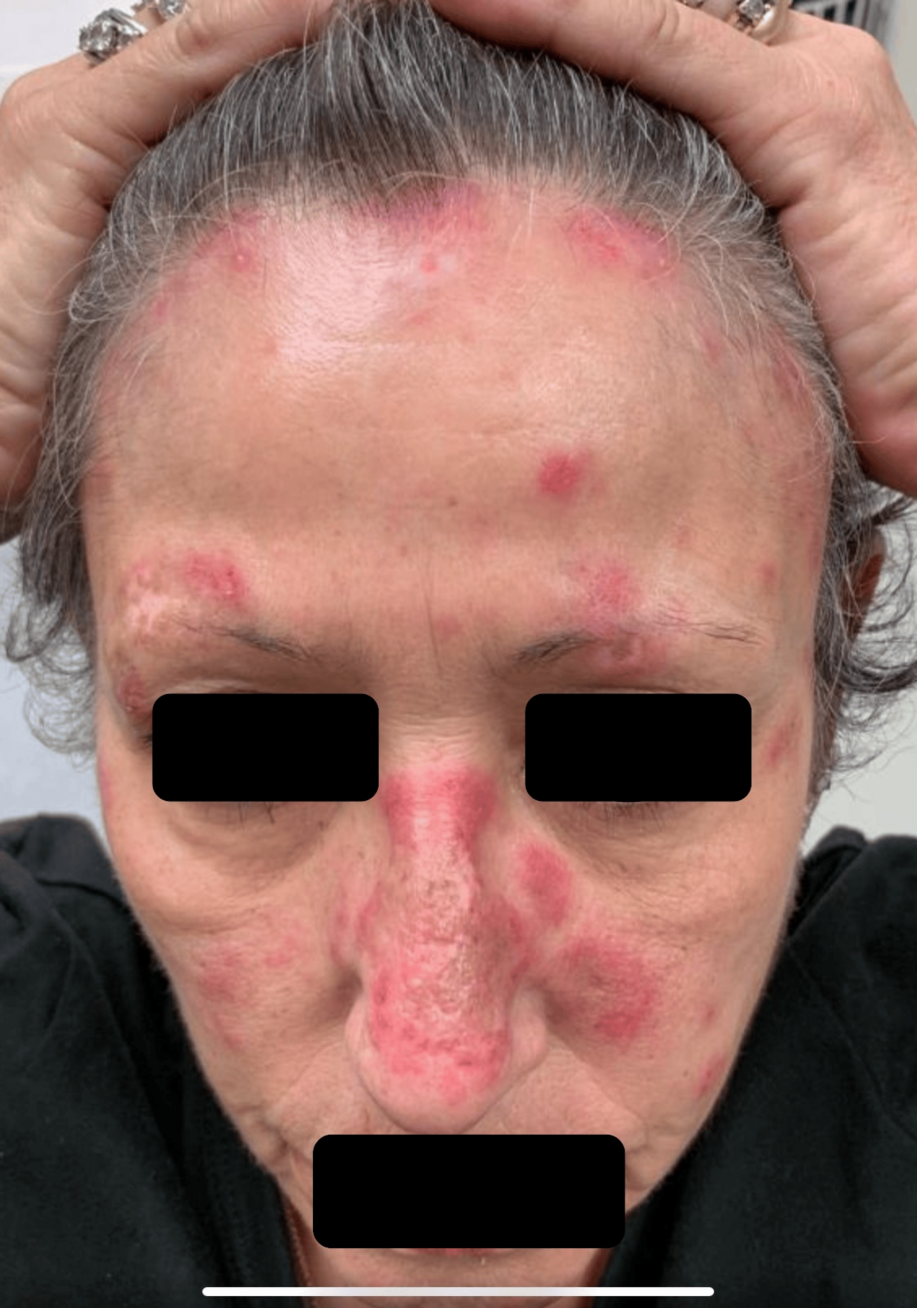
Erythematous papules and plaques on the face, frontal scalp, forehead, and eyebrows with areas of alopecia

**Figure 3:**
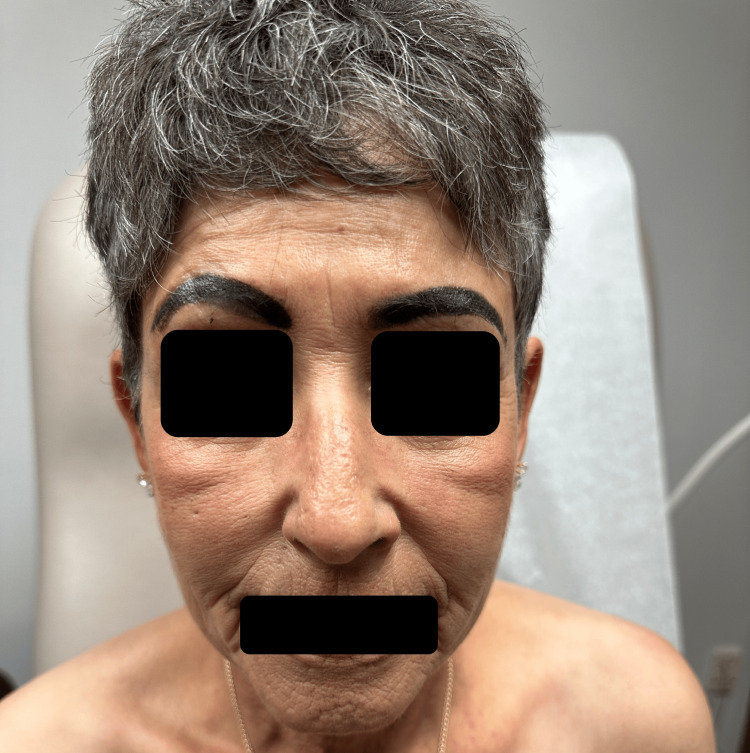
Resolution of lesions on the face and forehead at eight months of deucravacitinib therapy

**Figure 4:**
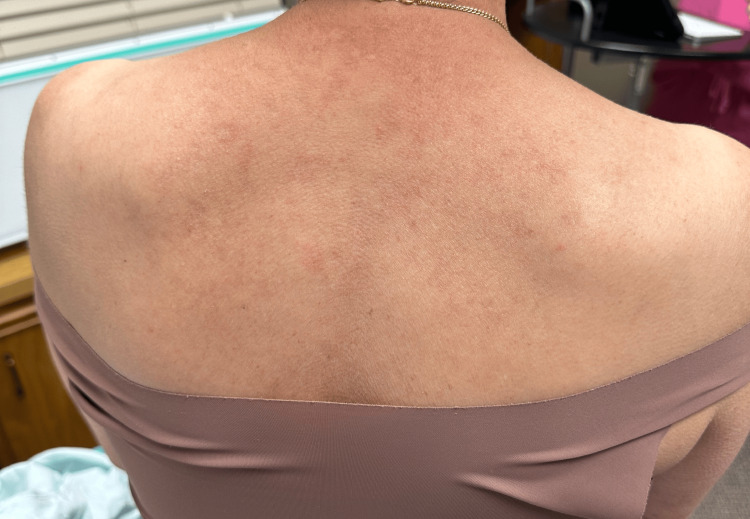
Resolutions of lesions on the upper back with areas of post-inflammatory hyperpigmentation at eight months of deucravacitinib therapy.

At a recent appointment, the patient was started on 6 mg daily of deucravacitinib. At this time, the patient presented with active erythematous plaques involving the upper back (Figure 1), midface, forehead, frontal scalp, and eyebrows with areas of alopecia (Figure 2). Deucravacitinib was continued for five months, during which the patient did not use oral prednisone, and minimal topical ruxolitinib cream was used. At this five-month follow-up appointment, the patient exhibited only post-inflammatory hypopigmentation in areas of previously active lesions on the face and back. Between the five- and eight-month follow-up appointments, the patient noted increased sun exposure in the summer months with no new lesions noted. After eight months, the patient had clinical resolution of all active lesions and continues to remain lesion-free (Figures 3, 4).

## Discussion

For many years, SCLE and DL have largely been managed with antimalarial therapy, most often hydroxychloroquine sulfate in doses of up to 200 mg twice daily. Patients resistant to antimalarials are often refractory to other treatment modalities, including systemic immunosuppressants such as methotrexate, mycophenolate mofetil, and azathioprine [1]. Often in these patients, multiple courses of systemic steroids and intralesional triamcinolone become the regimen of disease control, as was the case in our presented patient. 

Deucravacitinib is an allosteric inhibitor of the JAK/STAT pathway that functions by binding intracellular kinase. TYK2 may represent a novel method of disease control in treatment-resistant patients with chronic cutaneous lupus. Within lesional skin, type I interferons, including IFN-α/β, play a key role in immune response amplification, where they propagate the conversion of monocytes to dendritic cells. In turn, dendritic cells secrete IFN-α/β and are responsible for antigen presentation and priming of cell-mediated immunity. Deucravacitinib inhibits the propagation of the type I interferon signals as well as other cytokines, including IL-10, IL-12, and IL-23, and is already FDA approved for the treatment of psoriasis.

In the current literature, minimal reports of SCLE and DL have been treated with deucravacitinib, with only three cited in the literature at this time. In these cases, deucravacitinib was started due to recalcitrant disease and failure of multiple medications. Of known cases, two included starting deucravacitinib within three years of diagnosis with SCLE, and the third included a patient with a 30-year history of SCLE with concurrent palmoplantar pustulosis when starting deucravacitinib. All cases showed rapid improvement of active disease within four months of starting deucravacitinib [3-5].

In the first case, the treatment time of deucravacitinib was for one month in a patient with treatment-refractory SCLE presenting with symptoms of a malar rash, facial swelling, and erythematous scaly lesions on the back that worsened by sunlight. Like our patient, she was resistant to multiple treatment modalities, including oral prednisolone, hydroxychloroquine, methotrexate, and mycophenolate mofetil. This case did not follow up on the patient for longer than four weeks after starting deucravacitinib and the resolution of active lesions [3].

The second case included a patient with over 30 years of history of biopsy-proven discoid lupus erythematosus of the scalp and ears managed with hydroxychloroquine, topical fluocinolone oil, and intralesional triamcinolone. A recent increase in lesions of the scalp with associated alopecia necessitated the start of methotrexate and acitretin without improvement. Additional lesions of suspected palmoplantar pustular psoriasis that had been present for six months prompted the addition of deucravacitinib to her treatment regimen. The patient reported improvement of symptoms of pain and pruritus of her scalp discoid lupus lesions at one month with no additional follow-up [4].

The final case notes a patient with a history of a scaly rash on the right cheek of SCLE present for a year before presentation. The patient was initially treated with traditional regimens, including low-potency topical corticosteroids, desonide 0.05% cream, tacrolimus 0.1% ointment, hydroxychloroquine, methotrexate, and mycophenolate mofetil. After showing minimal improvement, the patient was started on deucravacitinib. At the four-month follow-up, her rash had cleared [5].

Our presented case will add to the limited literature of recalcitrant SCLE and DL treated successfully with deucravactinib. Our case represents the longest current follow-up period within the literature without recurrence for eight months. It is to be noted that deucravacitinib therapy for SLE patients is being studied with a recently completed phase 2 clinical trial and has shown to be better than placebo across measures of disease outcomes [2]. In the future, additional research and longitudinal studies of patients with cutaneous lupus are necessary to determine the long-term effectiveness of deucravacitinib. Given the success in patients resistant to traditional treatment regimens for cutaneous lupus, clinicians should consider deucravacitinib therapy.

## Conclusions

Subacute cutaneous lupus and DL represent a spectrum of chronic cutaneous lupus that presents cosmetic and psychological problems for affected patients without disease control. In cases where traditional treatment with antimalarials has failed, cutaneous lupus is often also resistant to alternative immunosuppressants and topical medications. A growing body of literature, including this case, suggests that targeting the JAK/STAT pathway and TYK2 intracellular kinase with deucravacitinib may prove beneficial in the management of this chronic disease. Further research and longitudinal study of patients with cutaneous lupus is needed to determine the long-term effectiveness of deucravacitinib.
